# Large-scale UK audit of blood transfusion requirements and anaemia in patients receiving cytotoxic chemotherapy

**DOI:** 10.1054/bjoc.1999.0883

**Published:** 1999-12-08

**Authors:** P J Barrett-Lee, N P Bailey, M E R O'Brien, E Wager

**Affiliations:** Velindre NHS Trust, Whitchurch, Cardiff, CF4 7XL, UK; Newcastle General Hospital, Newcastle, UK; Royal Marsden Hospital, London, UK; Janssen-Cilag Ltd

**Keywords:** anaemia, blood transfusion, antineoplastic agents, growth substances, erythropoietin

## Abstract

Cancer patients receiving cytotoxic chemotherapy often become anaemic and may require blood transfusions. A large-scale audit of patients with a variety of solid tumours receiving chemotherapy at 28 specialist centers throughout the UK was undertaken to quantify the problem. Data were available from 2719 patients receiving 3206 courses of cytotoxic chemotherapy for tumours of the breast (878), ovary (856), lung (772) or testis (213). Their mean age was 55 years (range 16–87). Overall, 33% of patients required at least one blood transfusion but the proportion varied from 19% for breast cancer to 43% for lung. Sixteen per cent of patients required more than one transfusion (7% for breast, 22% in lung). The mean proportion of patients with Hb < 11 g dl^−1^rose over the course of chemotherapy from 17% before the first cycle, to 38% by the sixth, despite transfusion in 33% of patients. Of the patients receiving transfusions, 25% required an inpatient admission and overnight stay. The most common symptoms reported at the time of transfusion were lethargy, tiredness and breathlessness. Further research is needed to evaluate the role of blood transfusions in patients receiving cytotoxic chemotherapy. © 2000 Cancer Research Campaign


British Journal of Cancer (2000) **82**(1), 93–97
© 2000 Cancer Research Campaign
Article no. bjoc.1999.0883

### Large-scale UK audit of blood transfusion requirements and anaemia in patients receiving cytotoxic chemotherapy

**PJ Barrett-Lee[1], NP Bailey[2], MER O'Brien[3] and E Wager[4,*]**

[1]Velindre NHS Trust, Whitchurch, Cardiff CF4 7XL, UK; [2]Newcastle General Hospital, Newcastle, UK; [3]Royal Marsden Hospital, London, UK, [4]Janssen-Cilag Ltd

**Summary** Cancer patients receiving cytotoxic chemotherapy often become anaemic and may require blood transfusions. A large-scale audit of patients with a variety of solid tumours receiving chemotherapy at 28 specialist centres throughout the UK was undertaken to quantify the problem. Data were available from 2719 patients receiving 3206 courses of cytotoxic chemotherapy for tumours of the breast (878), ovary (856), lung (772) or testis (213). Their mean age was 55 years (range 16–87). Overall, 33% of patients required at least one blood transfusion but the proportion varied from 19% for breast cancer to 43% for lung. Sixteen per cent of patients required more than one transfusion (7% for breast, 22% in lung). The mean proportion of patients with Hb < 11 g $dl^{-1}$ rose over the course of chemotherapy from 17% before the first cycle, to 38% by the sixth, despite transfusion in 33% of patients. Of the patients receiving transfusions, 25% required an inpatient admission and overnight stay. The most common symptoms reported at the time of transfusion were lethargy, tiredness and breathlessness. Further research is needed to evaluate the role of blood transfusions in patients receiving cytotoxic chemotherapy. © 2000 Cancer Research Campaign

**Keywords:** anaemia; blood transfusion; antineoplastic agents; growth substances; erythropoietin

Anaemia in cancer patients may be caused by the disease itself or the effects of treatments such as cytotoxic chemotherapy. Severe anaemia will increase the burden of treatment, contribute to fatigue and reduced quality of life and may even delay or limit further treatment. Blood transfusion is currently the most common form of treatment. The use of blood transfusions in patients undergoing oncological surgery or radiotherapy has been reviewed (Levine and Vijayakumar, 1993; Houbiers et al, 1995) but less has been published about patients receiving chemotherapy. We present the findings of a large-scale retrospective audit carried out at oncology centres across the UK which was designed to measure the prevalence of anaemia and the use of blood transfusions.

### METHODS

Retrospective audits of patients' notes were performed by specially trained nurses (employed specifically for this role by Janssen-Cilag Ltd) in collaboration with 28 participating oncology units between December 1996 and January 1998. Paper-based record collection was used at the first few centres to test the design of the forms but, once this was finalized, data were collected directly onto computer and, once rendered anonymous, were transmitted electronically to a third party (Innovex Ltd) for analysis. Data from each audit were presented confidentially to staff at the relevant centre. Aggregate, anonymized data were made available to the steering group of clinicians and to the sponsors (Janssen-Cilag Ltd).

*Received 28 September 1998*
*Revised 4 June 1999*
*Accepted 8 July 1999*

*Correspondence to:* PJ Barrett-Lee

Records from adult patients receiving cytotoxic chemotherapy for tumours of the breast, ovary, lung or testis were included in the audit. The concentration of haemoglobin prior to each cycle of chemotherapy was recorded (within-cycle measurements were not recorded). Details of blood transfusions (number of transfusions, number of units and need for overnight admission) were recorded for each cycle of chemotherapy.

Statistical significance was tested using parametric methods: $\chi^2$ tests were used to compare proportions of patients (e.g. those with anaemia or receiving transfusions), *t*-tests were used to compare averages (e.g. mean transfusion trigger), analysis of variance was used to test for a centre effect.

### RESULTS

Records from 2719 patients receiving 3206 courses of cytotoxic chemotherapy at 28 oncology centres were included in the audit. The patients had a mean age of 55 years and comprised 2052 women and 667 men. Patients' characteristics and diagnoses are shown in Table 1. Wide ranges of cytotoxic agents were prescribed but, for each tumour type one or two regimens predominated. The most common chemotherapeutic agents were: breast cancer, cyclophosphamide + 5-fluorouracil (5-FU) + methotrexate (CMF; 76% of patients), cisplatin + epirubicin + 5-FU (ECF; 16%); ovarian cancer, carboplatin (63%), adriamycin + cisplatin + cyclophosphamide (15%); lung cancer, cisplatin + mitomycin C + vinblastine (26%); adriamycin + cyclophosphamide + etoposide (ACE; 20%); testicular cancer, bleomycin + cisplatin + etoposide (BEP; 81%).

The proportion of patients with anaemia (defined as haemoglobin (Hb) < 11 g $dl^{-1}$) increased over the course of treatment

* Present address: Glaxo Wellcome, Greenford, Middlesex, UK

**Table 1** Patient characteristics

| Tumour type | Number of patients | Number of courses of chemotherapy | Mean age | (range) |
|---|---|---|---|---|
| All | 2719 | 3206 | 55 | (16–87) |
| Breast | 878 | 1053 | 51 | (24–87) |
| Ovary | 856 | 1071 | 59 | (21–87) |
| Lung | 772 | 832 | 61 | (28–84) |
| Testes | 213 | 250 | 34 | (16–76) |

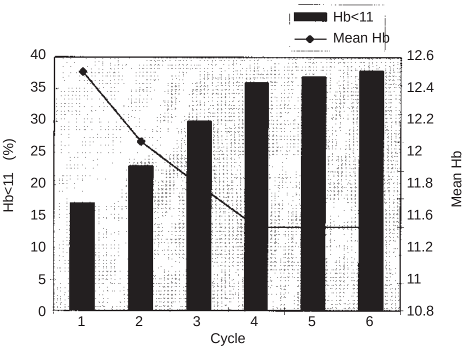

| Cycle | 1 | 2 | 3 | 4 | 5 | 6 |
|---|---|---|---|---|---|---|
| Hb<11 (%) | 17 | 23 | 30 | 36 | 37 | 38 |
| Mean Hb | 12.5 | 12.0 | 11.7 | 11.4 | 11.4 | 11.4 |
| N | 3101 | 3025 | 2791 | 2457 | 1903 | 1671 |

**Figure 1** Graph showing mean haemoglobin concentrations and the proportion of anaemic patients by chemotherapy cycle

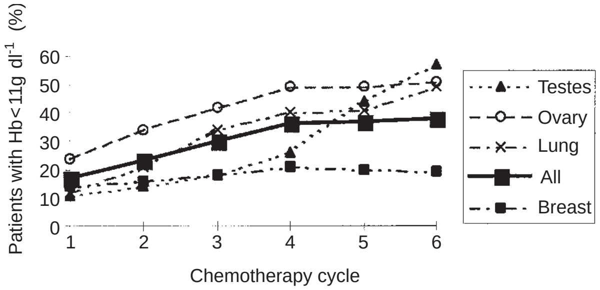

**Figure 2** Graph showing the proportion of patients with Hb < 11 g dl$^{-1}$ by tumour type and chemotherapy cycle

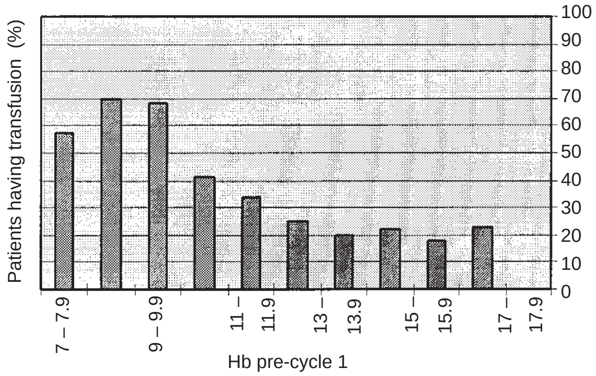

**Figure 3** Graph showing the proportion of patients receiving blood transfusions according to their prechemotherapy haemoglobin concentrations. (Patients with pre-treatment concentrations < 10 g dl$^{-1}$ were significantly more likely to receive transfusions than those with higher haemoglobin concentrations, $P < 0.001$.)

(Figure 1) but to different extents in the different tumour types (Figure 2). After the fourth course of chemotherapy, the proportion of anaemic patients was significantly higher in the ovarian and lung cancer groups than in patients with breast or testicular cancer ($P < 0.001$). By the sixth course of chemotherapy the proportion of patients with Hb < 11 g dl$^{-1}$ was significantly lower in the breast cancer group than the others ($P < 0.001$). The percentage of anaemic patients in the testicular cancer group rose sharply after the fourth cycle of chemotherapy but only a small proportion of patients received more than four cycles of chemotherapy ($n = 172$ at cycle 4 but 48 at cycle 5). Similarly, only a minority of patients with lung cancer (37%) received more than four cycles of chemotherapy whereas most patients with breast or ovarian cancer (64% and 78% respectively) received six cycles. On average, one in three patients received a blood transfusion at some time during cytotoxic treatment but the proportion varied between the different tumour types.

### Risk factors for transfusion

Patients with a pretreatment Hb concentration of < 11 g dl$^{-1}$ were significantly more likely to receive a transfusion than those who were not anaemic before chemotherapy. For example, in the first cycle, 24% of patients with pretreatment haemoglobin < 11 g dl$^{-1}$ received a transfusion while only 3% of other patients did ($P < 0.001$). The proportion of patients receiving transfusions increased markedly if the pretreatment Hb concentration was below 10 g dl$^{-1}$. The proportions of patients with pretreatment Hb of < 10 g dl$^{-1}$ receiving a transfusion at some time during their treatment were 100% (4/4) for testicular cancer, 75% (21/28) for lung cancer, 66% (31/47) for ovarian cancer and 60% (18/30) for breast cancer. For each tumour type this proportion was significantly higher than that for patients with pre-treatment haemoglobin concentrations above 10 g dl$^{-1}$ ($P \leq 0.03$ in all cases). However, only a minority of patients with pretreatment Hb < 10 g dl$^{-1}$ received a transfusion before their second cycle (15% for patients with ovarian cancer, 23% for patients with breast cancer). The overall proportion of patients receiving transfusions according to their pretreatment Hb levels is shown in Figure 3.

Patients with lung or ovarian cancer were significantly more likely to receive transfusions than those with breast or testicular tumours (42% for lung/ovarian vs 20% for breast/testicular, $P < 0.001$) (Table 2). The mean age of the patients who received transfusions was similar to that of the other patients.

Tumour stage was recorded in 1339 cases (410 patients with breast cancer, 166 with lung cancer, 632 with ovarian cancer and 131 with testicular cancer). Patients with metastatic breast, ovarian or testicular cancer were significantly more likely to receive a transfusion than patients with non-metastatic disease (44% vs 15% for breast $P = 0.002$, 64% vs 43% for ovarian $P < 0.001$, 73% vs 28% for testicular $P < 0.001$). The majority of lung cancer patients had non-small cell/unstaged disease and patients with small-cell tumours comprised only 21% of the group (166/772). However, those with non-metastatic small-cell lung cancer were

© 2000 Cancer Research Campaign

**Table 2** Blood transfusion requirements: proportions of patients requiring any transfusions and of those requiring more than one transfusion

| Tumour type | Patients requiring: Any blood transfusion | > 1 blood transfusion | Mean cycle 1 transfusion trigger (g dl$^{-1}$Hb) |
|---|---|---|---|
| All | 902 (33%) | 443 (16%) | 10.7 |
| Lung | 335 (43%) | 170 (22%) | 11.0[a] |
| Ovary | 347 (41%) | 179 (21%) | 10.5 |
| Testes | 51 (24%) | 29 (14%) | 9.6[b] |
| Breast | 169 (19%) | 65 (7%) | 10.6 |

[a] Significantly higher than mean for other groups combined ($P = 0.008$); [b]significantly lower than mean for other groups combined ($P = 0.009$).

**Table 3** Mean number of units of blood given per transfusion and details of in-patient admission solely for transfusion

| Tumour type | Mean units/ transfusion | % requiring in-patient admission | Mean length of stay (days) |
|---|---|---|---|
| All | 2.7 | 25 | 1.5 |
| Breast | 2.6 | 13 | 1.8 |
| Ovary | 2.7 | 36 | 1.5 |
| Lung | 2.8 | 22 | 1.3 |
| Testes | 3.1 | 14 | 1.3 |

**Table 4** Variation between audit centres in the proportion of patients receiving a blood transfusion and in the mean transfusion trigger (data from centres with at least ten patients with any tumour type)

| | | Breast | Lung | Ovary | Testes |
|---|---|---|---|---|---|
| % patients receiving transfusion in audit centres | Minimum | 3.2 | 18.2 | 31.4 | 6.8 |
| | Maximum | 97.5 | 89.9 | 100.0 | 46.5 |
| Mean Hb trigger for transfusion in audit centres | Minimum | 7.6 | 9.0 | 8.2 | 8.8 |
| | Maximum | 10.4 | 10.5 | 10.6 | 10.6 |

significantly more likely to receive a transfusion than those with metastatic small-cell tumours (49% vs 69%, $P = 0.01$).

### Trigger for transfusion

The transfusion ‘trigger’ (i.e. the mean Hb concentration at which a transfusion was given) was 10.7 g dl$^{-1}$ for all patients (range 7.2–16.1) in the first cycle but this fell to 9.9 g dl$^{-1}$ (range 6.3–14.0) by the sixth cycle. The mean transfusion triggers in the lung cancer group were significantly higher during both the first and sixth chemotherapy cycles than those for other cancers (11 vs 10.2 g dl$^{-1}$ ($P = 0.008$) in cycle 1 and 10.4 vs 9.7 ($P < 0.001$) in cycle 6). The first cycle transfusion trigger for patients with testicular cancer was significantly lower (9.6 g dl$^{-1}$) than that for

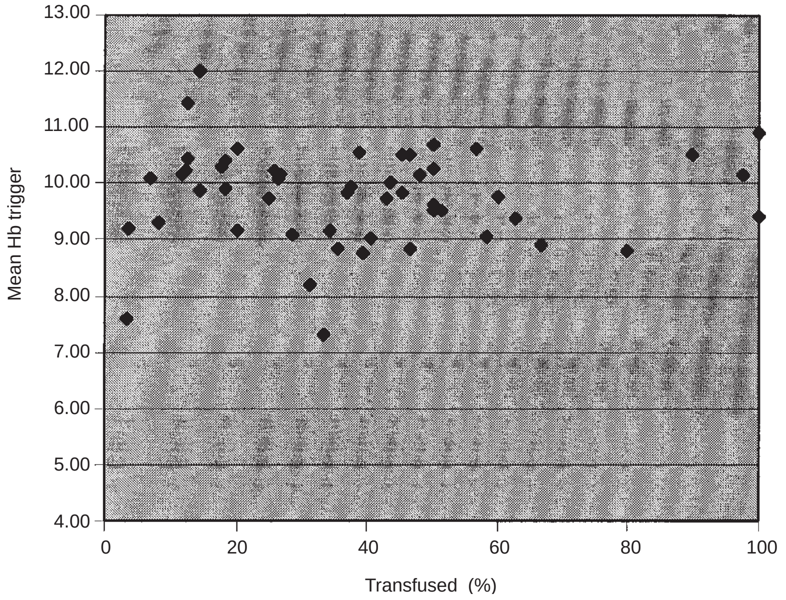

**Figure 4** Scatter plot of mean transfusion trigger (in g dl$^{-1}$ Hb) versus proportion of patients receiving transfusions for each tumour type in each centre (correlation coefficient = 0.003)

the other groups ($P = 0.009$). The mean number of units of blood given per transfusion and the proportion of patients who were admitted as in-patients solely for a transfusion are shown in Table 3.

Data from male and female patients with lung cancer were analysed separately to see if the mean transfusion triggers or proportion receiving transfusions was affected by gender. There were 452 men (59%) and 320 women (41%) in the lung cancer group but no difference in the mean transfusion triggers (10.4 for women vs 10.5 for men, $P = 0.3$).

### Variation by centre

The proportion of patients receiving a transfusion and the mean transfusion trigger showed significant differences between centres even when tumour type was controlled for ($P < 0.001$) (Table 4). There was no correlation between the mean transfusion trigger and the proportion of patients receiving a transfusion at each centre (correlation coefficient = 0.003) (Figure 4).

### Symptoms of anaemia

The symptoms for which transfusion was given were recorded for only 644 (40%) of the transfusion episodes. The most common symptoms recorded were lethargy (reported in 51% of notes in which any symptoms were mentioned), tiredness (42%), breathlessness (33%) and pallor (27%). The proportion of patients complaining of breathlessness was significantly higher in the lung cancer group than in the other groups (37% of those reporting symptoms compared with 20% in other groups, $P < 0.001$) while a significantly higher proportion of patients with cancer of the testis reported tiredness (57% vs 32%, $P < 0.001$). Because of the low number of patients for whom symptoms were recorded, we did not feel confident that patients with no record of symptoms in their notes were necessarily asymptomatic and it was therefore not possible to compare transfusion triggers in symptomatic and asymptomatic patients.

### DISCUSSION

The patients whose notes were included in the audit are probably representative of those receiving cytotoxic chemotherapy for the most common solid tumours in the UK. The preponderance of women is due to the fact that cancers of the breast and female reproductive organs are more likely to be treated with chemotherapy and/or have a higher incidence than the corresponding 'male' tumours such as prostate and testicular cancer. Patients with testicular cancer tended to be younger than those with other tumours. This is likely to affect their general level of health and the likelihood of having concomitant disorders that may exacerbate anaemia or its symptoms.

This survey found that, not unexpectedly, breathlessness was reported more frequently by patients with lung cancer and this may explain the observation that the trigger for transfusion among patients with lung cancer was higher than in other groups (i.e. the threshold for treating anaemia was lower). Studies from North America have shown similar findings and their authors have drawn similar conclusions to ours (Skillings et al, 1993). The observation that larger proportions of patients with cancer of the testis complain of tiredness than those with other tumour types was, perhaps, less predictable. However, this group also had the lowest trigger Hb concentrations. This may indicate that clinicians are more reluctant to give transfusions to younger patients with a good chance of recovery than to patients with a shorter life expectancy, unless their symptoms are severe.

Variations in the transfusion trigger between cancer types and across centres and the lack of correlation between transfusion trigger and the proportion of patients transfused suggest that low Hb per se is not routinely treated but that decisions about the need for transfusion are based on a combination of the Hb concentration and the presence of anaemia symptoms. Other authors have noted that the indication for blood transfusion is influenced by local policy (Dische, 1991) and may even vary within hospitals (Houbiers et al, 1995). Our findings also indicate considerable variation in transfusion practice between centres. Although the lower limits of normal Hb concentrations are usually taken as 12 g $dl^{-1}$ for women and 14 g $dl^{-1}$ for men, the figure of 10 g $dl^{-1}$ seems to be an arbitrary trigger point and many clinicians appear to wait until patients' haemoglobin concentrations fall 'into single figures' before giving a transfusion. If the aim of transfusion is to normalize Hb then transfusion triggers for men and women should be different but data from our lung-cancer patients showed no such difference. A review of radiotherapy and anaemia concluded that the threshold of 10 g $dl^{-1}$ is too low but that further trials were needed to determine whether a level of 12.5 g $dl^{-1}$, suggested by some other authors, was optimal (Dische, 1991). Our survey shows that, even when Hb concentrations are < 10 g $dl^{-1}$ before chemotherapy, only a minority of patients receive a blood transfusion before the start of the second chemotherapy course.

A significant proportion of patients were anaemic (Hb < 11 g $dl^{-1}$) and this increased after repeated cycles of treatment despite the fact that the majority of these patients also received at least one blood transfusion. Although this audit did not measure the duration of effect of transfusion, either on the Hb concentration or on the symptoms of anaemia, another audit of cancer patients in the palliative care setting has reported that the duration of subjective benefit may only be a few days (Finlay and Jenkins, 1997).

Other studies of anaemia in cancer have demonstrated that Hb levels correlate with patients' quality of life and functional ability (Henry and Abels, 1994; Leitgeb et al, 1994). In addition to these important aspects, anaemia may be associated with a worse prognosis and poorer response to treatment in patients undergoing radiotherapy for cancer (Dische, 1991; Levine and Vijaykumar, 1993). Evidence about the effects of blood transfusion on patients undergoing oncological surgery is conflicting and it is difficult to distinguish the effects of the anaemia itself, or its causes or other confounding factors with the unwanted effects of allogeneic blood transfusion (Houbiers et al, 1995). Our audit did not record response to chemotherapy or survival therefore we were not able to contribute to the debate about the effects of blood transfusion on these parameters but this would be an interesting question for future audits.

It is often difficult to predict which patients will develop anaemia and require treatment. Our findings suggest that the likelihood of receiving a transfusion is not related to the patient's age but does vary with the tumour type and stage. This is likely to be confounded by the different chemotherapy regimens used to treat different cancers and may be as much a function of the treatment as of the underlying disease. Other studies have shown that patients receiving platinum-based regimens frequently require transfusions (Cascinu et al, 1993). However, comorbidity from other diseases or direct effects of the tumour itself, as in lung

© 2000 Cancer Research Campaign cancer, may also affect the probability of a patient requiring a transfusion.

Treatment for anaemia in UK cancer patients usually involves blood transfusion. An economic study calculated the average cost of a transfusion of red cells to the National Health Service in 1994/5 to be £192 (Guest et al, 1998). The current audit suggests that 25% of cancer patients receiving transfusions were admitted to hospital for an average of 1.5 days. As the economic model reported above calculated the cost of 1 day's occupancy of a bed in a haematology ward at £247 (at 1994/5 prices) (Guest et al, 1998), this suggests that the average cost of transfusions for cancer patients will be considerably higher than £192. The current audit only recorded those stays that were solely due to transfusion and did not record cases in which patients' stay in hospital was prolonged because of transfusion or occasions when patients received several different treatments, which may be common in this population.

When considering the economic consequences of blood transfusion the direct and indirect costs to the patient and informal carers should also be borne in mind. This will include costs for travel to hospital, time off work, etc. and the intangible costs of the inconvenience and discomfort of receiving a transfusion.

A full analysis of the costs and benefits of available treatments should also include measurements of the patients' quality of life. Recombinant human erythropoietin (rHu-EPO) is one possible alternative to blood transfusions in some anaemic cancer patients. In clinical trials, patients receiving rHu-EPO have reported a higher quality of life than those receiving placebo plus conventional treatment with transfusions (Cascinu et al, 1993). However, rHu-EPO is not widely used in the UK because of its high acquisition cost. The socio-economic implications therefore need to be considered in any future research.

This audit has identified considerable variation between centres in their treatment of anaemia, and, in particular, a wide variation in the triggers for transfusion. Further information and research about the indications for blood transfusion, the role of alternative treatments such as erythropoietin, and their effectiveness in these patients are needed.

### CONCLUSIONS

This audit showed that a significant proportion of patients receiving cytotoxic chemotherapy will become anaemic and many of these will receive blood transfusions. The proportion of anaemic patients increases with repeated cycles of chemotherapy despite transfusions, suggesting that the duration of transfusion benefit may be short-lived. Triggers for transfusion vary, suggesting that low Hb levels are not routinely treated unless symptoms of anaemia become troublesome. Further research on the cost-effectiveness of blood transfusion and alternative treatments is required to ensure that scarce resources are used most effectively and quality of life is optimized for this group of patients.

### ACKNOWLEDGEMENTS

This audit was funded by Janssen-Cilag Ltd. Data analysis was performed by Innovex Ltd. We thank Victoria Burgin, Alison Cairns, Caroline Fitzgerald, Sarah Gould, Alison Harrison, Alison Jamieson and Sharon Lennon for collecting the audit data and Howard Cramer of Innovex for his work on data analysis. We thank all staff at the participating oncology centres that co-operated with the audit and helped with this project.

### REFERENCES

Cascinu S, Fedeli A, Luzi Fedeli S and Catalano G (1993) Cisplatin-associated anaemia treated with subcutaneous erythropoietin. A pilot study. *Br J Cancer* **67**: 156–158

Dische S (1991) Radiotherapy and anaemia – the clinical experience. *Radiother Oncol* Suppl **20**: 35–40

Finlay IG and Jenkins MJ (1997) Six-year audit of blood transfusion for hospice patients: simple criteria increase benefit. *Eur Assoc Palliative Care*

Guest JF, Munro V and Cookson RF (1998) The annual cost of blood transfusion in the United Kingdom. *Clin Lab Haematol* **20**: 111–118

Houbiers JGA, Busch ORC, Van de Watering LMG, Marquet RL, Brand A, Jeekel H and Van de Velde CJH (1995) Blood transfusion in cancer surgery: a consensus statement. *Eur J Surg* **161**: 307–314

Leitgeb C, Pecherstorfer M, Fritz E and Ludwig H (1994) Quality of life in chronic anemia of cancer during treatment with recombinant human erythropoietin. *Cancer* **73**: 2535–2542

Levine EA and Vijayakumar S (1993) Blood transfusion in patients receiving radical radiotherapy: a reappraisal. *Onkologie* **16**: 79–87

Skillings JR, Sridhar FG, Wong C and Paddock L (1993) The frequency of red cell transfusion for anemia in patients receiving chemotherapy. *Am J Clin Oncol* **16**: 22–25

© 2000 Cancer Research Campaign
